# Conditioned Media From Glial Cells Promote a Neural-Like Connexin Expression in Human Adipose-Derived Mesenchymal Stem Cells

**DOI:** 10.3389/fphys.2018.01742

**Published:** 2018-11-29

**Authors:** Debora Lo Furno, Giuliana Mannino, Rosalia Pellitteri, Agata Zappalà, Rosalba Parenti, Elisa Gili, Carlo Vancheri, Rosario Giuffrida

**Affiliations:** ^1^Department of Biomedical and Biotechnological Sciences, Section of Physiology, University of Catania, Catania, Italy; ^2^Institute of Neurological Sciences, National Research Council, Catania, Italy; ^3^Department of Clinical and Experimental Medicine, University of Catania, Catania, Italy

**Keywords:** human adipose mesenchymal stem cells, neural-like differentiation, connexin expression, glial conditioned media, olfactory ensheathing cells, Schwann cells

## Abstract

The expression of neuronal and glial connexins (Cxs) has been evaluated in adipose-derived mesenchymal stem cells (ASCs) whose neural differentiation was promoted by a conditioned medium (CM) obtained from cultures of olfactory ensheathing cells (OECs) or Schwann cells (SCs). By immunocytochemistry and flow cytometer analysis it was found that Cx43 was already considerably expressed in naïve ASCs and further increased after 24 h and 7 days from CM exposition. Cx32 and Cx36 were significantly improved in conditioned cultures compared to control ASCs, whereas a decreased expression was noticed in the absence of CM treatments. Cx47 was virtually absent in any conditions. Altogether, high basal levels and induced increases of Cx43 expression suggest a potential attitude of ASCs toward an astrocyte differentiation, whereas the lack of Cx47 would indicate a poor propensity of ASCs to become oligodendrocytes. CM-evoked Cx32 and Cx36 increases showed that a neuronal- or a SC-like differentiation can be promoted by using this strategy. Results further confirm that environmental cues can favor an ASC neural differentiation, either as neuronal or glial elements. Of note, the use of glial products present in CM rather than the addition of chemical agents to achieve such differentiation would resemble “more physiological” conditions of differentiation. As a conclusion, the overexpression of typical neural Cxs would indicate the potential capability of neural-like ASCs to interact with neighboring neural cells and microenvironment.

## Introduction

Mesenchymal stem cells (MSCs) are adult stem cells featuring self-renewal and differentiation abilities. They can typically give rise to mesodermal derivatives such as chondrocytes (Musumeci et al., 2011), osteocytes (Calabrese et al., 2015) or adipocytes (Lo Furno et al., 2013a) but, under appropriate conditions, they can also transdifferentiate into epithelial or neural cells (Zuk et al., 2002).

A neural differentiation of MSCs has been obtained by means of a variety of protocols. Safford et al. (2002) were able to induce adipose-derived MSCs (ASCs) into a neural phenotype by using a culture medium supplemented with valproic acid, butylated hydroxyanisole, insulin, and hydrocortisone. Several other supplements have been experimented thereafter, on MSCs of different sources (Krampera et al., 2007). To date, a growing body of literature indicates the possibility of a neural differentiation of ASCs. This differentiation is supported by the induction of typical neural marker, evaluated both at protein expression and mRNA levels (Goudarzi et al., 2018), as well as for their functional characteristics (Jang et al., 2010; Guo et al., 2016; Ching et al., 2018). Moreover, some transcription factors such as Pax6, NeuroD1, Tbr2, and Tbr1, associated to different stages of neurogenesis, were found overexpressed in ASCs after neural induction (Cardozo et al., 2012).

In our previous studies (Lo Furno et al., 2013b, 2018), a successful differentiation of ASCs toward neural-like elements was achieved by using a conditioned medium (CM) from cultures of Schwann cells (SCs) or olfactory ensheathing cells (OECs). This differentiation was documented by the increased expression of typical neural markers (Nestin, PGP9.5, MAP2, Synapsin I, GFAP).

In the present work, during a similar CM-promoted neural-like differentiation, the presence and/or the modification of the immunostaining of some connexins (Cxs) was investigated. In fact, Cxs are fundamental components of gap junctions which, though widely present in most tissues, play a crucial role in nervous system physiology. They allow direct communication between neurons and/or glial cells. Other than electrical signals, they mediate intercellular propagation of ions, second messengers and other small metabolites (Evans and Martin, 2002).

In adjacent cells, gap junctions characteristically consist of two opposed hemichannels (connexons), each made up of six Cxs (Söhl et al., 2005). Gap junctions consist of homotypic or heterotypic channels. In homotypic channels, both connexons are made by the same Cx subtype; in heterotypic channels, each connexon contains a different Cx subtype (Magnotti et al., 2011). Of note, they can connect cytoplasmic membranes of the same cell, forming autologous or reflexive gap junctions (Nualart-Marti et al., 2013). In addition, the presence of unpaired connexons (hemichannels) provides a communication device between the intra- and extra-cellular environments (Hartfield et al., 2011).

Neurons and glial cells of the mammalian brain are characterized by various Cx isoforms (Rozental et al., 2000; Rash et al., 2001; Vicario et al., 2017). Although with different cellular specificity, Cx32, Cx36, Cx43, and Cx47 are widely distributed.

Cx32 is expressed in the central nervous system (CNS) as well as in the peripheral nervous system (PNS). It is particularly found in oligodendrocytes and SCs, the two glial cells responsible for axon myelination (Mambetisaeva et al., 1999; Nualart-Marti et al., 2013).

Cx47 is characteristically expressed by oligodendrocytes (Parenti et al., 2010). It may exert compensatory effects when Cx32 is lacking. In fact, the loss of either Cx32 or Cx47 alone does not lead to evident alterations, whereas the absence of both Cxs significantly impairs axon myelination. Symptoms are characterized by gross tremors and tonic seizures, and death usually occurs after about 50 postnatal days (Menichella et al., 2003; Odermatt et al., 2003).

Cx43 is mainly present in astrocytes (Dermietzel et al., 1991; Iacobas et al., 2005), where it mediates ionic and metabolic exchanges within single or between neighboring cells. Through heterotypic Cx43/Cx47 gap junctions, astrocytes may be coupled with oligodendrocytes (Xu et al., 2017). Cx43 is also present in OECs (Barnett et al., 2001; Rela et al., 2010) and neural progenitors at early embryonic development stages (Duval et al., 2002).

Cx36 is typically expressed in neurons (Condorelli et al., 1998; Parenti et al., 2000, 2002; Rash et al., 2000). High levels of Cx36 have been detected in several regions of the CNS such as the inferior olive, cerebellum, hippocampus, hypothalamus and mammillary bodies (Condorelli et al., 2000). Although not exclusively, Cx36 is mainly found in GABAergic interneurons. This Cx is likely associated with the neuronal development, because its increased expression in several brain regions largely corresponds to interneuronal coupling in early postnatal weeks (Gulisano et al., 2000; Hartfield et al., 2011).

Herein, CM from OEC or SC cultures was used in the present investigation to promote a neural differentiation of ASCs. Both SCs and OECs are glial cells normally present in the nervous system, providing axon myelination. SCs are exclusively located in the PNS whereas OECs can also be found inside the CNS, where they support the continuous neurogenesis in the mammalian olfactory system (Ramón-Cueto and Avila, 1998). Both glial cells play an important role in axonal regeneration and remyelination (Sasaki et al., 2011; Gao et al., 2018). Their functional support is also provided by producing various growth factors and extracellular matrix molecules (Frostick et al., 1998; Pellitteri et al., 2001).

Connexin expression and their modifications in ASCs exposed to these conditioned media were evaluated by immunocytochemistry and flow cytometry analysis. The neural commitment of CM cultured ASCs was tested by the expression of Nestin, PGP9.5 and GFAP.

Data were gathered after 24 h and 7 days of culture and compared to control ASCs. Results suggest that, considering the pattern of Cx profile modification, a neural fate was promoted in ASCs under these conditions.

## Materials and Methods

### Preparation of Rat OEC-CM

Experimental procedures were carried out according to the Italian Guidelines for Animal Care (D.Lgs 26/2014), and the European Communities Council Directives (2010/63/EU), and were approved by the ethics committee of the University of Catania (Organismo Preposto al Benessere Animale, OPBA; Authorization n. 174/2017-PR). All efforts were made to minimize animal suffering and to reduce the number of animals used.

As previously described, OECs were isolated from olfactory bulbs of 2-day-old rat pups (Pellitteri et al., 2009). Briefly, the bulbs were removed and cold dissected (+4°C) in Leibowitz L-15 medium (Sigma-Aldrich, Milan, Italy). Subsequently, they were digested by collagenase (Invitrogen, Milan, Italy) and trypsin (Sigma-Aldrich) in Minimum Essential Medium-H (MEM-H, Sigma-Aldrich). Enzymatic activity was stopped by adding Dulbecco’s Modified Eagle’s Medium (DMEM) supplemented with 10% fetal bovine serum (FBS, Sigma-Aldrich; DMEM/FBS). The antimitotic agent cytosine arabinoside (10^-5^ M) was added to reduce the number of dividing fibroblasts. In order to increase OEC proliferation, bovine pituitary extract was added. Finally, cells were incubated at 37°C in fresh DMEM/FBS, which was replaced twice a week. OEC-CM was collected 24–48 h after reaching confluence. It was filtered to remove debris and detached cells, aliquoted and stored at -20°C until further use.

### Preparation of Rat SC-CM

To harvest SCs, sciatic nerves of 2-day-old rat pups were removed and treated with collagenase and trypsin in DMEM (Pellitteri et al., 2006). They were then mechanically dissociated by trituration and filtered through a 150 μm nylon mesh. After centrifugation, cells were resuspended and plated in 25 cm^2^ flasks containing fresh DMEM/FBS. The antimitotic agent cytosine arabinoside (10^-5^ M) was added to reduce the number of dividing fibroblasts.

After 24–48 h from confluence, SC-CM was collected, filtered to remove debris and detached cells, aliquoted and stored at -20°C until further use.

### Human ASC Cultures

Adipose tissue was harvested from healthy young donors undergoing liposuction procedures at the Cannizzaro Hospital, Catania (Italy). Written informed consent was given by all donors to use the lipoaspirate for experimental procedures, which were carried out in accordance with the Declaration of Helsinki (2000). The protocol was approved by the local ethics committee (Comitato etico Catania1; Authorization n. 155/2018/PO).

First, the lipoaspirate was digested by collagenase (Invitrogen) in DMEM before the following steps, as already described (Lo Furno et al., 2013b). Briefly, after centrifugation at 1,200 rpm for 10 min, the pellet was resuspended in PBS and filtered through a 100-μm nylon cell strainer (Falcon BD Biosciences, Milan, Italy). Cells were then plated in T75 culture flasks (Falcon BD Biosciences) with DMEM/FBS and 1% MSC growth supplement (MSCGS; ScienCell Research Laboratories, Milan, Italy). After 24 h of incubation at 37°C with 5% CO_2_, the growth medium was replaced to remove non-adherent cells. When about 80% of confluence was reached, cells were trypsinized and plated for the subsequent procedures.

As in previous works, the MSC nature of cells was tested by immunocytochemistry and flow cytometry. In particular, it was verified that cells were immunopositive for typical MSC markers (CD 44, CD 73, CD 90, and CD 105) and immunonegative for typical hematopoietic stem cell markers (CD 14, CD 34, and CD 45). Moreover, their multipotential differentiation toward chondrocytes, osteocytes and adipocytes was previously verified (Calabrese et al., 2015).

For the specific purpose of this investigation, some cultures were incubated with OEC-CM or SC-CM. Some others served as controls, maintained in DMEM/FBS. One sample of each culture type was stopped after 24 h, whereas other samples were kept for 7 days. At each time point, fluorescence immunocytochemistry and flow cytometry procedures were carried out for evaluating Cx expression. Additional samples were used to test the expression of neural markers (Nestin, PGP9.5 and GFAP). To evaluate cell morphology and growth rate, some cell cultures were stained with hematoxylin.

### ASC Immunocytochemistry

Immunostaining procedures were carried out following a protocol previously described (Lo Furno et al., 2013b). Briefly, cells were fixed with 4% paraformaldehyde and incubated for 30 min with a solution of PBS containing normal goat serum (5%; Sigma-Aldrich) and Triton (0.1%; Sigma-Aldrich). They were then exposed overnight at 4°C to primary antibodies: anti-Cx32 (1:100; made in mouse; Novex), anti-Cx36 (1:100; made in mouse; Novex), anti-Cx43 (1:100; made in rabbit; Sigma-Aldrich), anti-Cx47 (1:100; made in mouse; Novex), anti-Nestin (1:100; made in rabbit; Abcam, Cambridge, United Kingdom), anti-PGP9.5 (1:100; made in rabbit; Novus Biologicals, Milan, Italy), anti-GFAP (1:100; made in mouse; Abcam). The following day, cells were incubated for 1 h at room temperature with secondary antibodies conjugated to different fluorochromes: FITC-conjugated goat anti-rabbit (1:500; Abcam) or Cy3-conjugated goat anti-mouse (1:500; Abcam).

In some experiments, a double immunostaining procedure was carried out to verify the immunopositivity for Cx and neural markers in the same cells. In particular, some ASC cultures were tested for Nestin and Cx36; some others for PGP9.5 and Cx32. Immunostaining was revealed by FITC-conjugated and Cy3-conjugated secondary antibodies. As a final step, DAPI staining (10 min) was carried out to visualize cell nuclei. In some samples, the primary antibody was omitted to verify the specificity of immunostaining.

### ASC Flow Cytometry

After trypsinization, cells were fixed (2% paraformaldehyde in PBS for 20 min at 4°C) and permeabilized (1% Triton in PBS for 5 min at 4°C). Cells were then incubated at room temperature for 30 min with 1% BSA to block non-specific sites, and for 1 h with the primary antibodies. The same antibodies mentioned above were used, at the same dilutions. Successively, cells were exposed for 1 h to goat anti-mouse or goat anti-rabbit FITC-conjugated secondary antibodies (1:200; Abcam). A Coulter Epics Elite ESP flow cytometer (Coulter, Miami, FL, United States) was used. For each sample, a maximum of 10,000 forward and side scatter gated events were collected. Excitation wavelength was 488 nm and fluorescence monitoring was at 525 nm wavelength. Mean fluorescence intensity (MFI) values were calculated and recorded automatically.

### Statistical Analysis

Quantitative data of percentages of positive cells were gathered from three experiments. They are expressed as mean ± standard deviations. Statistical analysis [Student’s *t*-test for paired and unpaired data; variance analysis (ANOVA)] was performed using the statistical software package SYSTAT, version 11 (Systat Inc., Evanston, IL, United States). Tukey’s ‘Honest Significant Difference’ method was used as a *post hoc* test. A difference was considered significant at *P* < 0.05.

## Results

**FIGURE 1:**
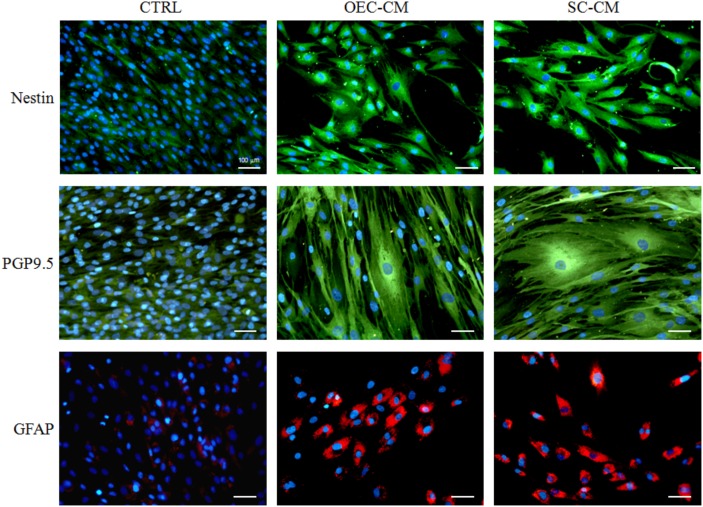
Photomicrographs of ASC cultures after 7 days of growth in basal medium (CTRL), in OEC-CM or in SC-CM. Compared to controls, immunostaining for Nestin (upper row), PGP9.5 (middle row) and GFAP (lower row) is substantially increased in CM treated cultures. ASC, adipose-derived stem cell; OEC-CM, conditioned medium from olfactory ensheathing cells; SC-CM, conditioned medium from Schwann cells. Magnification: 20×; Scale bars: 100 μm.

The stem cell profile of ASCs was verified by immunocytochemistry and flow cytometry. In accordance with previous studies (Calabrese et al., 2015), cells were immunopositive for typical MSC markers (CD 44, CD 73, CD 90, and CD 105) and immunonegative for typical hematopoietic stem cell markers (CD 14, CD 34, and CD 45). Moreover, as reported recently (Lo Furno et al., 2018), an ASC neural-like phenotype by using OEC-CM or SC-CM was here confirmed by the increased immunopositivity for Nestin, PGP9.5, and GFAP (Figure 1).

### Cell Growth and Morphology

**FIGURE 2:**
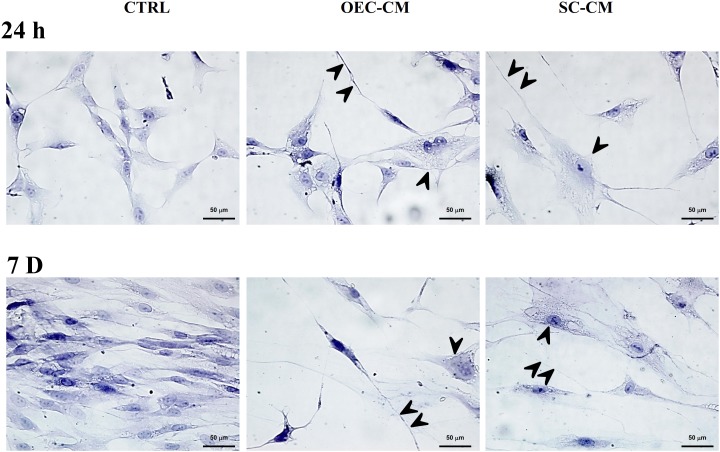
Photomicrographs of hematoxylin stained adipose-derived stem cells (ASCs) cultured in basal medium (CTRL, left), in OEC-CM (middle) or in SC-CM (right). The three conditions are shown after 24 h (upper row) and 7 days (lower row) of growth. Typically, control ASC cultures show fibroblast-like cells, much more numerous after 7 days of growth. Cells cultured in either OEC-CM or SC-CM were less numerous, especially at 7 days. They are characterized by bigger cell bodies (arrowheads). In OEC-CM cultures, elongated cytoplasmic branches were frequently observed (double arrowheads). OEC-CM, conditioned medium from olfactory ensheathing cells; SC-CM, conditioned medium from Schwann cells. Magnification: 40×; Scale bars: 50 μm.

After 24 h of growth in the basal MSC medium, ASCs exhibited a typical fibroblast-like morphology (Figure 2). Those cultured in OEC-CM or in SC-CM were bigger and showed a more complex cytoplasmic shape. At 7 days, ASCs in control cultures were much more numerous, showing a similar shape to that observed at 1 day. Instead, cells cultured in either OEC-CM or SC-CM were more scattered, featuring bigger and much more complex cell bodies; long and thin cytoplasmic branches were often detectable, more evident in OEC-CM treated cells.

### Connexin Expression

**FIGURE 3:**
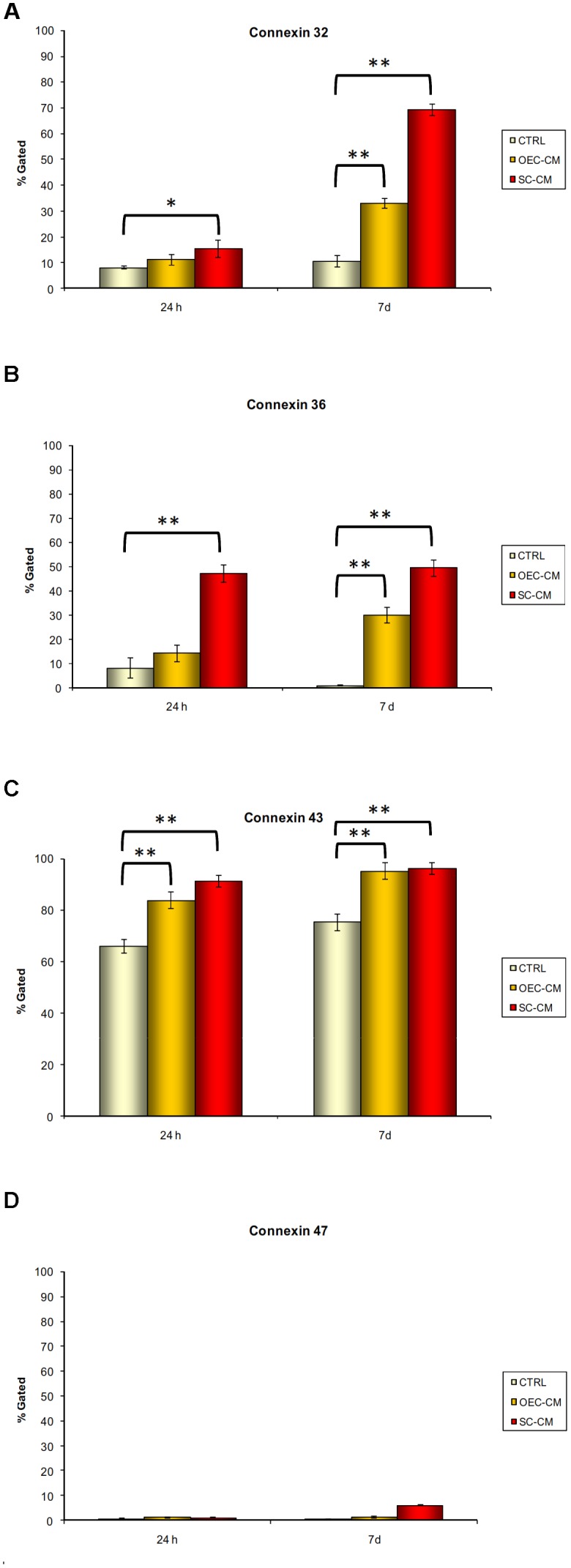
Percentages of immunopositive cells for connexin 32 **(A)**, 36 **(B)**, 43 **(C)**, and 47 **(D)** after 24 h and 7 days of culture. Data were gathered from three independent experiments where adipose-derived stem cells were cultured in basal medium (controls), in conditioned medium from olfactory ensheathing cells (OEC-CM) or in conditioned medium from Schwann cells (SC-CM). Significant differences between CM treated cultures and controls are indicated: ^∗^*P* < 0.05, ^∗∗^*P* < 0.001.

Immunofluorescence and Flow cytometry was used to evaluate the pattern of cellular Cx expression at 24 h and 7 days of culture. In particular, three conditions were investigated: (a) control ASCs, kept in the basal medium, (b) ASCs cultured in OEC-CM, or (c) SC-CM. Overall, observations at the fluorescence microscope were consistent with flow cytometry outcomes. Quantitative data were gathered from three independent experiments. They are summarized in Table 1, where mean values and standard deviation of positive cells and MFI are reported for each condition at each stage of signal detections. Percentages of positive cells in the different conditions are also reported in the histograms of Figure 3, where significant differences between CM treated cultures and controls are highlighted.

**Table 1:** Flow cytometry data showing effects of OEC-CM or SC-CM on Cx expression in ASCs after 24 h and 7 days of culture.

		24 h			7 days		
		Controls	OEC-CM	SC-CM	Controls	OEC-CM	SC-CM
Cx32	% positive cells	8.12 ± 0.5	11.29 ± 2.1	15.46 ± 3.5	10.54 ± 2.3	33.10 ± 2.0	69.46 ± 2.2
	MFI	1.2 ± 0.4	1.4 ± 0.3	1.6 ± 0.5	1.4 ± 0.3	2.3 ± 0.2	2.8 ± 0.4
Cx36	% positive cells	8.26 ± 4.1	14.36 ± 3.3	47.2 ± 3.5	0.92 ± 0.2	30.02 ± 3.1	49.47 ± 3.3
	MFI	1.8 ± 0.3	1.9 ± 0.2	2.9 ± 0.3	1.5 ± 0.2	1.9 ± 0.4	2.3 ± 0.2
Cx43	% positive cells	66.10 ± 2.5	83.90 ± 3.2	91.29 ± 2.3	75.46 ± 3.2	95.31 ± 3.1	96.14 ± 2.3
	MFI	2.6 ± 0.5	3.3 ± 0.3	4.6 ± 0.4	2.6 ± 0.6	5.0 ± 0.4	5.4 ± 0.2
Cx47	% positive cells	0.77 ± 0.2	1.39 ± 0.3	1.31 ± 0.2	0.59 ± 0.1	1.33 ± 0.4	6.31 ± 0.3
	MFI	1.1 ± 0.2	1.2 ± 0.3	1.3 ± 0.2	1.2 ± 0.1	1.7 ± 0.4	1.6 ± 0.4

*All data are expressed as means and standard deviations (Means ± SDs) of three values. Adipose-derived stem cells (ASCs) cultured in basal medium (controls), in conditioned medium from olfactory ensheathing cells (OEC-CM) or conditioned medium from Schwann cells (SC-CM). Cx, connexin; MFI, mean fluorescence intensity.*

**FIGURE 4:**
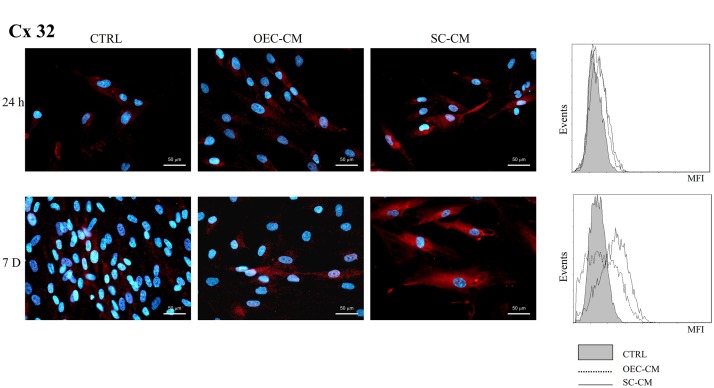
Photomicrographs of Cx32 immunostaining **(A)** of ASC cultures in basal medium (CTRL), in OEC-CM or in SC-CM. Flow cytometry data are shown in **(B)**. Data were collected after 24 h (upper row) and after 7 days (lower row). More evident increases are noticeable for SC-CM treated ASCs after 7 days. ASC, adipose-derived stem cell; OEC-CM, conditioned medium from olfactory ensheathing cells; SC-CM, conditioned medium from Schwann cells. Magnification: 40×; Scale bars: 50 μm.

Immunopositivity for Cx32 (Figure 4) was present in control ASC cultures both at 24 h and 7 days, although in a low percentage of cells (8% and 10%, respectively). In CM cultures, only slight increases could be observed at 24 h, whereas significantly higher percentages were found after 7 days, especially for SC-CM vs. OEC-CM treatment (69% and 33%, respectively). MFI values in CM cultures were correspondently higher than those in control ASCs. Again, more pronounced increases were found in SC-CM samples at 7 days.

**FIGURE 5:**
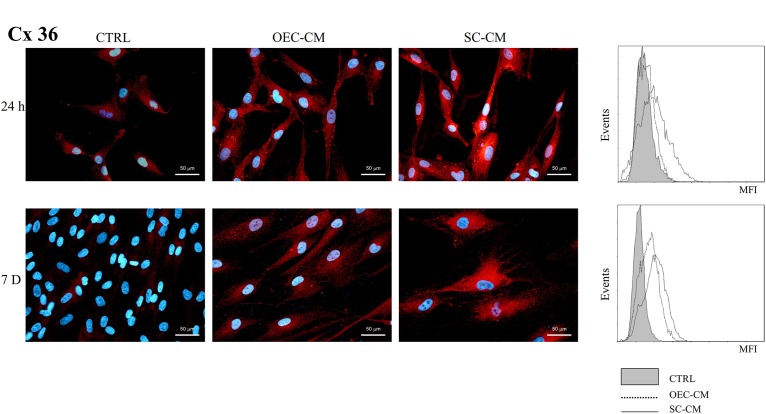
Photomicrographs of Cx36 immunostaining **(A)** of ASC cultures in basal medium (CTRL), in OEC-CM or in SC-CM. Flow cytometry data are shown in **(B)**. Data were collected after 24 h (upper row) and after 7 days (lower row). More evident increases are visible for SC-CM treated ASCs, already after 24 h. ASC, adipose-derived stem cell; OEC-CM, conditioned medium from olfactory ensheathing cells; SC-CM, conditioned medium from Schwann cells. Magnification: 40×; Scale bars: 50 μm.

Cx36 immunopositivity (Figure 5) was modestly present in control ASC cultures at 24 h (8%), and it was almost undetectable at 7 days (less than 1%). In OEC-CM cultures, only slight increases were found at 24 h (14%), whereas significantly higher percentages were observed after 7 days (30%). More marked effects were detected in SC-CM samples. In these cases, the incidence of ASC immunopositive cells, both at 24 h and 7 days, was almost 50%. In addition, SC-CM was able to induce effects faster (already visible at 24 h), whereas OEC-CM effects were more evident after a longer period (7 days). MFI values were correspondently higher in SC-CM cultures.

**FIGURE 6:**
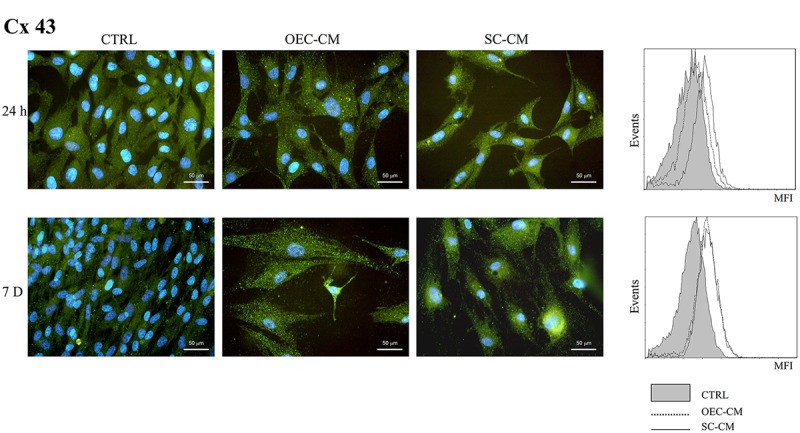
Photomicrographs of Cx43 immunostaining **(A)** of ASC cultures in basal medium (CTRL), in OEC-CM or in SC-CM. Flow cytometry data are shown in **(B)**. Data were collected after 24 h (upper row) and after 7 days (lower row). Immunoreactivity was evident already in control ASCs and further increased following OEC-CM or SC-CM treatment. ASC, adipose-derived stem cell; OEC-CM, conditioned medium from olfactory ensheathing cells; SC-CM, conditioned medium from Schwann cells. Magnification: 40×; Scale bars: 50 μm.

A considerably high incidence of Cx43 immunopositive cells (Figure 6) was present in control ASC cultures, both at 24 h and 7 days (about 70%). The treatment with OEC-CM or SC-CM evoked further increases of this Cx expression (above 90%). Flow cytometry data confirmed that MFI values, already considerable in control cultures, were even higher in CM samples.

**FIGURE 7:**
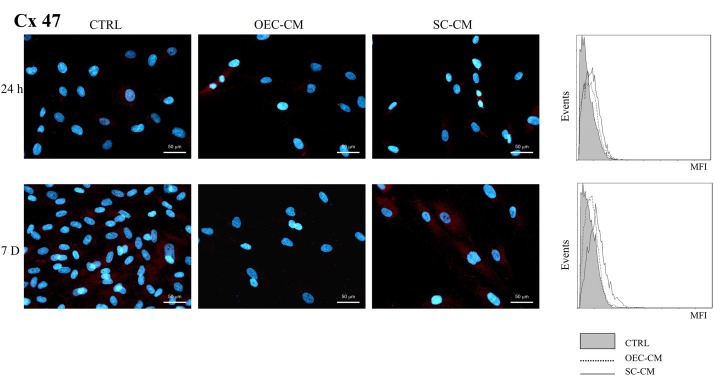
Photomicrographs of Cx47 immunostaining **(A)** of ASC cultures in basal medium (CTRL), in OEC-CM or in SC-CM. Flow cytometry data are shown in **(B)**. Data were collected after 24 h (upper row) and after 7 days (lower row). Immunoreactivity was virtually absent in all conditions. ASC, adipose-derived stem cell; OEC-CM,: conditioned medium from olfactory ensheathing cells; SC-CM, conditioned medium from Schwann cells. Magnification: 40×; Scale bars: 50 μm.

Almost undetectable levels of Cx47 immunoreactivity (Figure 7) were present in control ASC cultures, both at 24 h and 7 days (less than 1%). Exposition to OEC-CM or SC-CM induced only very slight, if any, effects. The greatest increase, although of modest entity (up to 6%), was observed only after 7 days of SC-CM treatment. It can be assumed that this Cx, virtually absent in control ASCs, was not modified in all the conditions tested.

**FIGURE 8:**
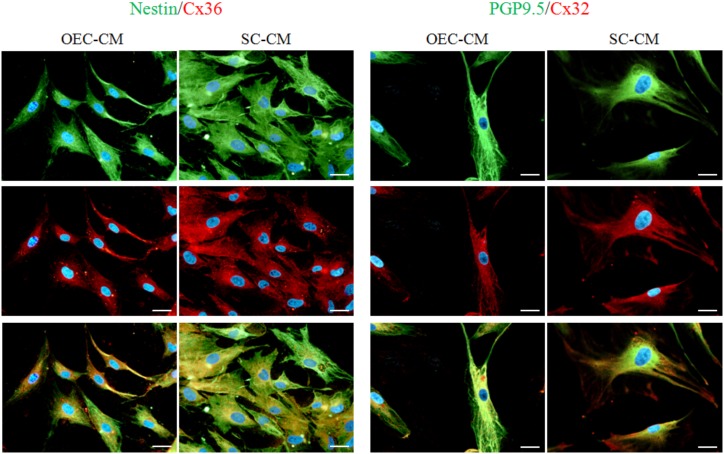
Photomicrographs of double labeled ASC cultures exposed to OEC-CM or to SC-CM for 7 days. In the two columns on the left, Nestin immunostaining (green, upper row) was combined with Cx36 (red, middle row). In the two columns on the right, PGP9.5 immunostaining (green, upper row) was combined with Cx32 (red, middle row). Note that the same cells are immunopositive both for neural markers and Cxs (merge in lower row). ASC, adipose-derived stem cell; OEC-CM, conditioned medium from olfactory ensheathing cells; SC-CM, conditioned medium from Schwann cells. Magnification: 40×; Scale bars: 50 μm.

Double labeling experiments allowed us to verify that Cxs and neural markers may indeed be expressed by the same cells. Some examples are illustrated in Figure 8, where Nestin and PGP9.5 immunostaining was combined with Cx36 and Cx32 immunoreactivity, respectively.

## Discussion

Results obtained in the present investigation support previous studies in which a neural-like differentiation of ASCs was promoted by conditioned media from OECs or SCs (Lo Furno et al., 2018). In fact, various Cxs, typical of neurons or glial cells, were found overexpressed by using a similar protocol.

In the absence of CM treatment, basal levels of Cx43 were considerably high; Cx47 was virtually absent; Cx32 and Cx36 were modestly found at 24 h and decreased at 7 days of culture. This decreased Cx expression is probably due to a reduced differentiation ability if ASCs are kept in the basal medium. The treatment with glial-derived conditioned media induced different degrees of modifications in the expression of the various Cxs, except for Cx47, whose immunostaining remained undetectable. In synthesis, high basal levels of Cx43 were further improved and significant increases were observed for Cx32 and Cx36.

Indeed, many of the Cxs tested can be found in tissues other than the nervous system. However, it is likely that only neurons or glial cells may exhibit this Cx combination. On the other hand, this interpretation is corroborated by the increased expression of neural markers such as Nestin, PGP9.5, and GFAP, and double labeling immunostaining. These findings are in keeping with a previous work where, by using the same protocol, an increased expression of various neural markers was observed, either when conspicuously present (Nestin and PGP9.5) or weakly expressed (GFAP, MAP2 and Synapsin I) in ASC control cultures (Lo Furno et al., 2018).

The increased Synapsin expression already suggested the possibility to establish synaptic connections with other neural cells. The overexpression of various Cxs here observed further supports this interactive potential. Present in virtually every neural cell type, Cxs are essential for the constitution of gap junctions, and play an important role in brain homeostasis. They are crucial for cell-to-cell communication, as well as for interactions between cells and the surrounding microenvironment.

Cx32 is particularly important in myelinating glial cells, where it mainly establishes reflexive gap junctions (Nualart-Marti et al., 2013). In doing so, these junctions provide faster radial cytoplasmic communication between the several layers of myelin down to the axon membrane, thus ensuring nutritional support and functional integrity. Cx43 is the main Cx subtype in astrocytes. By homotypic/heterotypic coupling between astrocytes and/or oligodendrocytes, it contributes to the formation of the so-called “panglial syncytium” (Rash et al., 1997; Magnotti et al., 2011). This glial network helps to stabilize membrane potential by absorption and removal of extracellular potassium ions released during action potential firing. Cx36 in neuronal gap junctions is essential for the formation of electrical synapses which are key elements for the electrotonic coupling between neurons and the synchronization of membrane potential (Condorelli et al., 2000; Nagy et al., 2018).

Altogether, our results indicate that high basal levels and increases of Cx43 expression suggest a strong predisposition of ASCs toward an astrocyte differentiation, as also suggested by high levels of GFAP previously reported (Lo Furno et al., 2018). On the other hand, the lack of Cx47 would indicate a lower propensity to differentiate into oligodendrocytes, at least by using the protocol adopted here. This kind of differentiation, however, cannot be ruled out, if using different strategies. Finally, observed increases of Cx32 and Cx36 would suggest a potential ASC differentiation toward neurons or SC.

Except for Cx32, whose increased expression was more pronounced in SC-CM cultures, comparable effects were observed for the other Cxs using each CM. This is probably due to the similarity of the two glial cell types, which play a similar functional role and are characterized by a similar immunophenotype. Similarly to astrocytes, SCs and OECs express GFAP (Bianchini et al., 1992; Pellitteri et al., 2010). Both SCs and OECs are characterized by their immunopositivity for S-100 and P75 ^NTR^ (Chung et al., 2004; Pellitteri et al., 2009). Since they largely produce a similar variety of cytokines/growth factors, it can be assumed that these factors are responsible for the observed modifications. In this regard, it should be underlined that the same culture medium was used both for control ASCs and for each of the two glial cells. Therefore, any CM-induced effect would not be related to different medium supplements, but only to glial products present in the CM.

Although the identification of these molecules was not the purpose of the present work, it is well established that both OECs and SCs are able to produce several growth factors (Kocsis et al., 2009; Gao et al., 2018) such as nerve growth factor (NGF), brain-derived neurotrophic factor (BDNF), glial cell derived neurotrophic factor (GDNF), ciliary neurotrophic factor (CNTF), and fibroblast growth factor (FGF). These factors are known to promote a neural differentiation of various cell types. Positive effects have been described for BDNF on survival and differentiation of neurons and neural stem cells, likely through upregulation of tropomyosin receptor kinase (Trk) B, phosphoinositide 3-kinase (PI3K), AKT and β-catenin (Li et al., 2017). CNTF and FGF2 were able to enhance the proliferation of Muller glia-derived progenitor cells (MGPCs), likely through the activation of Jak/Stat signaling pathway (Todd et al., 2016). A neural-like differentiation of BM-MSC has been promoted by GDNF (Ma et al., 2016). A stimulatory effect on Cx expression was also described (Cushing et al., 2005). For example, an overexpression of Cx43 was induced on PC12 cells by NGF through its receptor Trk A. Several pathways may be responsible for these effects, such as extracellular signal-regulated kinases (ERK-1/2) and protein kinase C (PKC). An increased expression of Cx 43 was also observed after treatment of C6 glioma cells with CNTF (Ozog et al., 2002).

Another hypothesis to explain CM-induced effects may take into account the presence in each CM of extracellular vesicles (EVs) and exosomes released by glial cells. EVs and exosomes have been recently recognized as intercellular messengers which can transfer mRNAs, microRNAs, proteins and lipids to recipient cells (Bátiz et al., 2016). If this is the case, ASC expression of Cx might result from the direct transfer of mRNA or proteins by EVs and exosomes to ASCs. Interestingly, this transfer likely involves exosomal Cx43, able to modulate the interaction and transfer of information between exosomes and acceptor cells (Soares et al., 2015). For example, this hypothesis would explain why SC-CM better stimulates Cx32 expression. In fact, Barnett et al. (2001) demonstrated that Cx32 protein was present in SCs but not in OECs, whereas Cx43 mRNA levels were similarly detected for both types of glial cells. According to other hypotheses, soluble factors bound to EVs/exosomes may also be responsible for intercellular communication mechanisms. In fact, it has been shown that multiple functional responses may be induced by signaling pathways which involve EV-associated cytokines (Cossetti et al., 2014). Probably, multiple mechanisms are involved in eliciting the final outcome. Further investigations are needed to elucidate whether different effects are exerted by different CM components.

Since high percentages of immunopositive cells were found in some instances both for typical glial and neuronal Cxs, it is reasonable to assume that two or more Cxs are simultaneously expressed in the same cells observed here. This event can, however, be explained if one takes into account that they might still be at early stages of neural differentiation. In fact, it has been reported that in neural progenitor cells both neuronal and glial markers still coexist (Wei et al., 2002; Kempermann et al., 2004; Vinci et al., 2016). Presumably, as the differentiation progresses, the expression of some Cxs would be favored while some others would be downregulated, likely on the basis of microenvironmental cues. For example, during embryonic development, Cx36 expression becomes restricted to neuronal cells, while Cx43 expression becomes restricted to astrocytes (Sadowska et al., 2009; Hartfield et al., 2011).

## Conclusion

In conclusion, this work and previous investigations confirm that a neural-like differentiation of ASCs can be achieved without adding to the culture medium chemical agents which could exert toxic effects. This seems of particular interest for potential therapeutic applications in the field of regenerative medicine, because newly differentiated neurons or glial cells would represent a valuable tool for healing PNS injuries (Xie et al., 2017; Ching et al., 2018) or for the treatment of chronic neurodegenerative disorders affecting the CNS.

Indeed, many encouraging results have been obtained worldwide not only *in vitro* but also in preclinical experiments and in clinical trials. In particular, better outcomes have been reported *in vivo* after administration of previously neural-induced elements rather than naïve stem cells. However, most studies attribute stem cell-induced progress to the paracrine production of cytokine/growth factors rather than to a real differentiation in functional nerve cells. In this context, every effort should be made to define differentiation protocols able to provide more permanently differentiated neurons or glial cells, whose target after administration would be to mend a disrupted brain circuitry or a loss of myelination. The use of conditioned media may help to develop some of these strategies because they mimic, in a more physiological way, the nervous tissue microenvironment in which neurons and glial cells normally work.

## Author Contributions

DL, GM, and RG wrote the paper, designed and performed the experiments. RPe prepared the conditioned media and discussed experimental results. AZ and RPa contributed with their connexin expertise and provided essential reagents. EG and CV performed the flow cytometry analysis. All authors read and approved the final version of the manuscript.

## Conflict of Interest Statement

The authors declare that the research was conducted in the absence of any commercial or financial relationships that could be construed as a potential conflict of interest.
